# Pre-treatment of soil X-ray powder diffraction data for cluster analysis

**DOI:** 10.1016/j.geoderma.2018.09.044

**Published:** 2019-03-01

**Authors:** Benjamin M. Butler, Andrew M. Sila, Keith D. Shepherd, Mercy Nyambura, Chris J. Gilmore, Nikolaos Kourkoumelis, Stephen Hillier

**Affiliations:** a The James Hutton Institute, Craigiebuckler, Aberdeen AB15 8QH, UK; b World Agroforestry Centre (ICRAF), P.O. Box 30677-00100 GPO, Nairobi, Kenya; c School of Chemistry, University of Glasgow, Glasgow G12 8QQ, UK; d Department of Medical Physics, School of Health Sciences, University of Ioannina, 45110 Ioannina, Greece; e Department of Soil and Environment, Swedish University of Agricultural Sciences (SLU), SE-75007 Uppsala, Sweden

**Keywords:** Soil mineralogy, X-ray powder diffraction, Cluster analysis

## Abstract

X-ray powder diffraction (XRPD) is widely applied for the qualitative and quantitative analysis of soil mineralogy. In recent years, high-throughput XRPD has resulted in soil XRPD datasets containing thousands of samples. The efforts required for conventional approaches of soil XRPD data analysis are currently restrictive for such large data sets, resulting in a need for computational methods that can aid in defining soil property – soil mineralogy relationships. Cluster analysis of soil XRPD data represents a rapid method for grouping data into discrete classes based on mineralogical similarities, and thus allows for sets of mineralogically distinct soils to be defined and investigated in greater detail. Effective cluster analysis requires minimisation of sample-independent variation and maximisation of sample-dependent variation, which entails pre-treatment of XRPD data in order to correct for common aberrations associated with data collection.

A 2^4^ factorial design was used to investigate the most effective data pre-treatment protocol for the cluster analysis of XRPD data from 12 African soils, each analysed once by five different personnel. Sample-independent effects of displacement error, noise and signal intensity variation were pre-treated using peak alignment, binning and scaling, respectively. The sample-dependent effect of strongly diffracting minerals overwhelming the signal of weakly diffracting minerals was pre-treated using a square-root transformation. Without pre-treatment, the 60 XRPD measurements failed to provide informative clusters. Pre-treatment via peak alignment, square-root transformation, and scaling each resulted in significantly improved partitioning of the groups (*p* < 0.05). Data pre-treatment via binning reduced the computational demands of cluster analysis, but did not significantly affect the partitioning (*p* > 0.1). Applying all four pre-treatments proved to be the most suitable protocol for both non-hierarchical and hierarchical cluster analysis. Deducing such a protocol is considered a prerequisite to the wider application of cluster analysis in exploring soil property – soil mineralogy relationships in larger datasets.

## Highlights

• A 2^4^ factor analysis design used to determine an appropriate pre-treatment protocol for cluster analysis of soil XRPD data.
• Alignment, square-root transformation and scaling of the data each significantly improve group partitioning (*p* < 0.05).
• Binning causes no significant improvement in group partitioning (*p* < 0.1), but reduces computational cost of cluster analysis.
• The determined pre-treatment protocol is valid for hierarchical and non-hierarchical cluster analysis of soil XRPD data.

## 1 Introduction

X-ray powder diffraction (XRPD) is a widely employed method in the study of complex mineral mixtures, with the varied mineral assemblages of soils providing particularly apposite examples (Bish, 1994; Dixon and Schulze, 2002). Conventional approaches to the assessment of soil mineralogy by XRPD typically involve a first stage of identification of the minerals present and a subsequent stage that seeks to quantify the relative abundance of the different minerals identified in the soil. In such a conventional approach the first stage of identification is typically made by an iterative process using reference databases and monographs that tabulate data for the diffraction patterns of different minerals that may be encountered (ICDD, 2016; Brindley and Brown, 1980; Dixon and Schulze, 2002; Harris and White, 2008). This tabulated reference data usually records peak positions in terms of their ‘d-spacings’ in Ångstrom (or nanometers) together with their relative intensities (0–100%). Despite the availability of automated search match procedures and other software tools to aid the identification stage, round robin evidence suggests that the process still relies heavily on the experience of the analyst to correctly identify (Raven and Self, 2017) the minerals in samples like soils.

Compared to mineral identification, the next conventional step of mineral quantification is widely acknowledged as a much more complex task (Omotoso et al., 2006). Quantification seeks to relate variation in the intensity of either individual mineral peaks or of the full patterns (i.e. all peaks) of each mineral to its concentration in the sample, usually expressed in weight %. Such quantitative analyses are most often made on multiple samples, so that variation in mineral abundance can be compared from one soil sample to another and ultimately interpreted in relation to soil properties and functions that are dependent on soil mineralogy. Again the procedures that may be used to perform quantitative mineralogical analyses of soils rely heavily on the experience of the analyst to ensure that the results obtained are reliable and fit for purpose (Raven and Self, 2017).

The data of modern XRPD methods which all these procedures require is a precisely measured digital diffraction pattern typically comprised of discrete ‘Bragg’ diffraction peaks varying in intensity (y), expressed for example in counts, along an experimental axis (x) usually expressed in degrees 2*θ*. The ‘Bragg’ peaks from crystalline mineral phases rise above a background, which may also include diffuse scattering from X-ray amorphous phases. For example organic matter and volcanic glass can be common amorphous phases in many soils (Dixon and Schulze, 2002).

In recent years the availability of digital XRPD soil data has increased, and attempts are now being made to generate datasets containing thousands of spatially referenced XRPD measurements [e.g. those collected for the National Soil Inventory of Scotland (NSIS) and the Africa Soil Information Service (AfSIS)]. Since many soil properties are closely related to soil mineralogy (Butler et al., 2018; Newman, 1984), such datasets in combination with computational data analysis represent unique opportunities to advance the understanding of the role of soil minerals in governing or influencing many soil properties, processes and functions.

In data-rich cases like NSIS (Butler et al., 2018) and AfSIS (Towett et al., 2015), computational methods of XRPD data analysis become particularly attractive because they are time-efficient and do not necessarily require any classical expert interpretation of the XRPD patterns, at least not until the final stages of such an analysis. This may seem like the analysis is initially disconnected from the crystallographic origins of the data, but this is the foundation of data-driven, or digital, approaches to soil mineralogy (Butler et al., 2018; Hillier and Butler, 2018). Cluster analysis is one such ‘digital’ approach that can aid the interpretation of large XRPD datasets by classifying the data into discrete clusters that can be more manageably interpreted and described in their groups (Barr et al., 2004a, Barr et al., 2004b, Barr et al., 2004c, Barr et al., 2004d; Gilmore et al., 2004). Many clustering methods exist, including the fuzzy-c-means algorithm (Bezdek et al., 1984) that has been considered particularly appropriate for the heterogeneous nature of soils (Viscarra Rossel et al., 2016), and hierarchical clustering that is currently implemented in the DIFFRAC.EVA software (Bruker) for XRPD data analysis. For soil XRPD data, a rational cluster should be comprised of soils with similar mineralogy (i.e. minimised intra-group variation), whilst separate clusters should reflect distinct soil mineralogies (i.e. maximised inter-group variation).

In effect, the application of approaches such as cluster analysis to digital XRPD data collected from soil samples allows the diffraction patterns to be treated simply as digital signatures which encode information on soil mineral (peaks) and amorphous components (background). Subsequent to cluster analysis, identification and quantification may then be more efficiently introduced to understand how differences in soil mineralogy have influenced the clusters observed and how they relate to soil property data.

### 1.1 Pre-treatment of XRPD data for cluster analysis

**Fig. 1:**
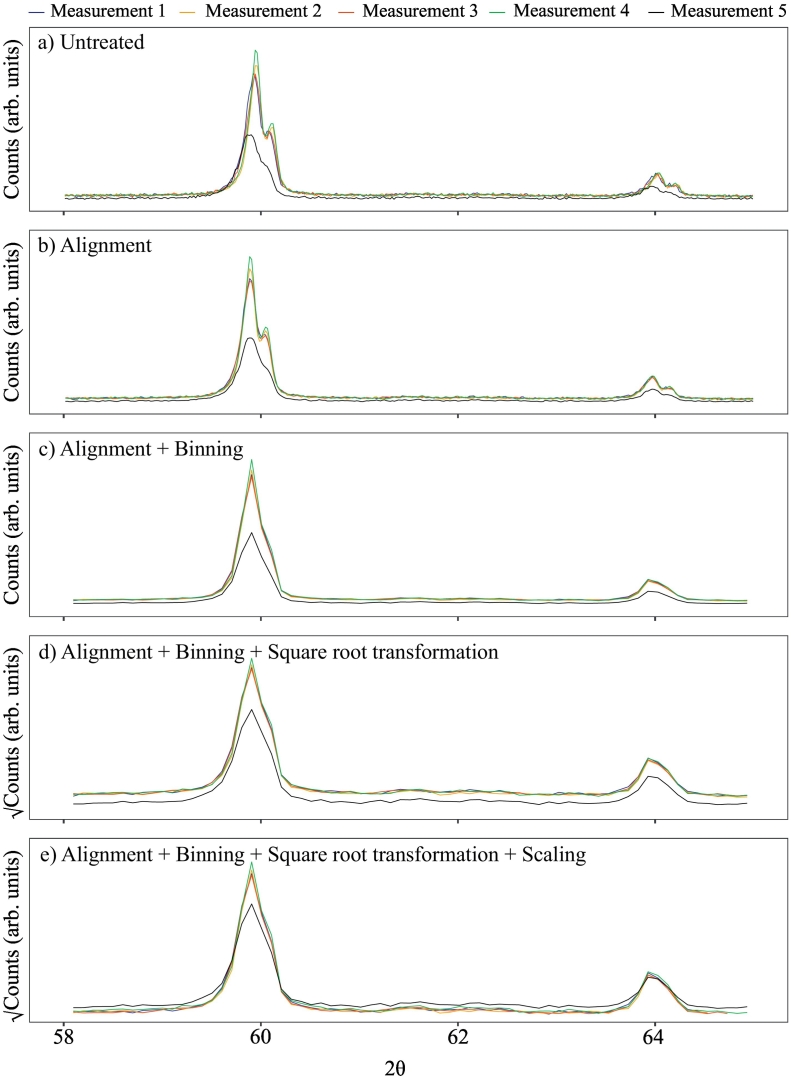
Effects of sequential pre-treatment steps on XRPD data illustrated using data from a single soil sample measured once by five different personnel. The 2*θ* range has been reduced to 58–65° to aid comparison between pre-treatments. Untreated data (a) display variations in peak alignment, noise, and signal intensity. Pre-treatment via alignment (b) results in suitably aligned peaks, and causes a slight smoothing of the data due to a linear spline interpolation used to harmonise the aligned data to the same 2*θ* scale (Section 2.3). Subsequent binning of the data (c; bin width = 5) acts to further reduce the noise whilst retaining sufficient mineralogical information, and simultaneously acts as a form of data reduction. Subsequent pre-treatment by square root transformation (d) re-scales the data so that minor peaks become emphasised relative to major peaks. Lastly, subsequent pre-treatment by scaling (e; mean centering) corrects for most of the variation in signal intensity between samples. (For interpretation of the references to colour in this figure legend, the reader is referred to the web version of this article.)

Measurement reproducibility, i.e. precision, plays a critical role in facilitating effective cluster analysis. It is therefore crucial to appreciate that the precision of XRPD measurements may be commonly affected by several experimental and specimen related factors that bear no relation to compositional differences between samples (Jenkins, 1989). Such factors may alter peak positions, relative and absolute intensities, and signal to noise ratios. Some of these variations may be further emphasised and potentially become more problematical when samples are prepared by multiple personnel, as is often the case in the collection of larger datasets. The aim of data pre-treatment prior to cluster analysis is to minimise these experimental and specimen related factors and promote computation of suitable clusters. Here we evaluate how peak alignment, noise, sample composition, and count intensities can affect XRPD data (Fig. 1).

Peak positions in XRPD data commonly shift in response to small variations in specimen height in the instrument, the so called ‘specimen displacement error’. An example of this error is illustrated in Fig. 1a. Even seemingly small misalignments between peaks can hinder the comparison of XRPD data by multivariate methods such as principal component analysis (Andersson and Hämäläinen, 1994; Arneberg et al., 2007), and hence reduce the effectiveness of cluster analysis. One approach to deal with such peak shifts is to use a mineral with essentially invariant peak positions as an internal standard (e.g. the common mineral quartz), resulting in well aligned data (Fig. 1b).

Noise in XRPD data relates to small fluctuations in response and detection, and to some extent is linked to the signal intensity and counting statistics (i.e. signal to noise ratios). Many approaches are available to reduce noise in XRPD data including moving averages and Savitsky-Golay filtering (Savitsky and Golay, 1964). Alternatively, binning data (combining a fixed number of adjacent data points into one combined variable) can be used to reduce noise whilst also acting as a method of data reduction that can be advantageous for computationally intensive exercises (Wehrens, 2011). A suitable bin width should be chosen that reduces noise but retains sufficient information within the peak shapes and intensities (Fig. 1c).

When based on principal component scores (Viscarra Rossel et al., 2016), cluster analysis of soil XRPD data has potential to be influenced by the signal of ‘strong’ diffractors, which is dependent upon sample mineralogy. Examples of strong diffractors include the omnipresent soil mineral quartz, which can overwhelm the less intense signal of weaker diffractors (e.g. feldspars and clay minerals) or minor but potentially important soil minerals (e.g. iron oxides). Because the information value related to what may appear as minor peaks can exceed that associated with intense peaks, by applying logarithmic or nth root transformations, skewness of the data can be reduced as the intensity of the strongest peaks relative to weaker peaks is rescaled. As outlined in Arneberg et al. (2007), logarithmic transforms modify linear correlations within data, whereas nth root transforms preserve any perfect linear correlations but reduce correlations in regions of partial correlation. Pre-treatment by nth root transform is therefore considered here to be most suitable for XRPD data (Fig. 1d).

Lastly, variations in signal intensities can also affect the outcome of cluster analysis. Such variations can be caused by several instrumental factors, including X-ray tube degradation, power settings, and choice of scan-time. Differences in signal intensities due to such factors may be scaled to comparable values by scaling methods such as mean centering (Wehrens, 2011; Viscarra Rossel et al., 2006), which subtracts the mean from all values in a sample vector of XRPD count intensities (Fig. 1e).

Further to the four factors outlined above, preferred orientation, or specimen texture, can be particularly troublesome in XRPD data since it alters the relative intensity of a mineral's Bragg peaks. Preferred orientation effects can be especially pronounced for minerals with prominent cleavage planes, which would include most clay minerals, feldspars, and carbonates for example. It can also be a very operator dependent issue, since it occurs during the loading of the specimen into its holder. Whilst the effects of preferred orientation cannot be accounted for using data pre-treatment, it can be eliminated during sample preparation using methods such as spray drying (Hillier, 1999) but in cases where this method is not available the specimen preparation protocol must be demonstrably reproducible (Zhang et al., 2003).

The aim of this present study was to assess the requirement for and effectiveness of data pre-treatment methods for minimising the influence of the experimental and specimen aberrations on soil XRPD cluster analysis. Here, a 2^4^ factorial design was used to assess the effects of four data pre-treatment protocols (alignment, binning, square-root transformation, and scaling) on the clustering of soil XRPD data primarily using the fuzzy-c-means algorithm. The factorial design employed all possible combinations of the four pre-treatments, allowing examination of individual effects as well as potential interactions between them. Ultimately the investigation is used to outline the most appropriate data pre-treatment protocol for soil XRPD cluster analysis using a combination of objective measures for group variance, number of clusters and cluster validity. The study is viewed as a necessary prelude to the application of cluster analysis to large datasets of soil XRPD patterns, such as NSIS and AfSIS, and to exploiting their value in the increasingly data-driven field of digital soil analysis.

## 2 Materials and methods

### 2.1 Soil samples

Soils were sourced from 10 of the 60 AfSIS ‘sentinel’ sites spread across Sub-Saharan Africa (Vågen et al., 2010). Each sentinel site covers 10 × 10 km, divided into 16 grid cells of equal size. Each grid cell contains 10 randomly selected plots. One composite topsoil (0–20 cm) sample from each of the 16 grid cells at each site was selected for analysis, yielding a dataset of 160 samples measured by XRPD (see Section 2.2). For this investigation, 12 soils were then selected from this dataset (*n* = 160) using the Kennard Stone algorithm (applied to untreated XRPD measurements) to provide a range of mineralogically distinct diffractograms (Kennard and Stone, 1969).

Although the mineralogical composition of the samples is not considered explicitly in the results of this study, it is useful to mention that the samples cover a wide range of mineralogical compositions that are commonly observed in the complete AfSIS data set (Towett et al., 2015). Specifically, phase identification using the Powder Diffraction File database (ICDD, 2016) and subsequent quantification by a full pattern summation method (Omotoso et al., 2006) indicates that the minerals/mineral groups encountered across the set of twelve samples and estimates of their range of relative abundance in weight percent include: quartz (13–93), feldspars (0–55), amphibole (0–3), epidote (0−12), ilmenite (0–15), goethite (0–15), hematite (0–8), gibbsite (0−13), mica/illite (0–13), kaolin (3–64), and expandable/mixed-layer (0–40) clay minerals.

### 2.2 X-ray powder diffraction

Subsampling for XRPD analysis was conducted by coning and quartering. Subsamples were then prepared for XRPD by McCrone milling 3 g of sieved (<2 mm) and air-dried soil for 12 min in ethanol. Excess ethanol was removed by centrifugation and each sample re-suspended in 1.5 ml of hexane. The samples were then oven dried at 80°C before being disaggregated and ground by hand in a mortar and pestle before passing through a 250 μm sieve. Each sample (Section 2.1) was loaded onto the instrument by five different personnel, and analysed, one of these runs being the original AfSIS data collection. Loading was carried out by loosely filling the sample holders with the finely ground powders, before flattening the surface with the sharp edge of a razor blade, with personnel instructed to take care to apply minimum pressure and to avoid shearing motion. The combination of methodical milling and loading was designed to produce samples with appropriate particle statistics and to minimise preferred orientation (Zhang et al., 2003) since the most effective procedure of spray drying the samples (Hillier, 1999) was not available in the AfSIS lab.

After loading, XRPD data were collected on a Bruker desktop D2 PHASER diffractometer, with Ni-filter, Cu-Kα radiation with the X-ray tube operated at 30 kV and 10 mA. The beam was collimated using a 0.6 mm divergence slit, a 1 mm anti-scatter slit and a 2.5 mm Soller slit. Samples were rotated continuously at 15 rpm during data collection over the angular range of 3 to 75°2*θ*, counting for 96 s per 0.02° step with a Lynxeye position sensitive detector.

### 2.3 Pre-treatments and factorial design

A 2^4^ factorial design [based on that used in Arneberg et al., 2007 for pre-treatment of mass spectrometry data] was performed on the dataset of 60 diffractograms (12 soils, each measured 5 times). The 4 pre-treatments were selected to correct for 2*θ* shifts (alignment), noise/data reduction (binning), overwhelming contributions from strong diffractors [using the square-root of the intensities, Kourkoumelis, 2013] and count intensity variation (scaling). The design allowed examination of individual pre-treatment effects on subsequent analysis (Section 2.5) as well as potential interactions between them. The 16 runs of the factorial design encompassed peak alignment (+1) versus no peak alignment (−1), binning (+1) versus no binning (−1), square-root transform (+1) versus no square-root transform (−1), and scaling (+1) versus no scaling (−1). The 16 factorial combinations are provided in Table 1. The same 16 combinations were also performed with data that had been subjected to de-noising using a discrete wavelet transform (Nason, 2016), but no noticeable effect on group variance or clustering was observed, and the results are therefore not presented.

**Table 1:** The 2^4^ factorial design applied to the XRPD dataset. A value of +1 denotes use of the pre-treatment, and a value of −1 denotes no use. *F* (derived from MANOVA, Section 2.5) represents division of the inter-group variance by the intra-group variance. *n*_clust_ is the optimum number of clusters obtained by maximising the partition coefficient in the fuzzy-c-means clustering. *ARI* is the Adjusted Rand index, computed as a measure of cluster validity (Section 2.5). The ‘F16-rerun’ factor relates to analysis that follows the protocols of F16, but with substitution of two samples (identified as having poor counting statistics) with re-analysed XRPD data.

ID	Alignment	Binning	Square-root	Normalisation	*F*	*n*_clust_	*ARI*
F1	−1	−1	−1	−1	29.61	2	0.04
F2	+1	−1	−1	−1	61.92	2	0.04
F3	−1	+1	−1	−1	30.90	2	0.04
F4	+1	+1	−1	−1	88.37	2	0.04
F5	−1	−1	+1	−1	66.72	2	0.07
F6	+1	−1	+1	−1	284.79	11	0.70
F7	−1	+1	+1	−1	70.71	2	0.07
F8	+1	+1	+1	−1	283.94	12	0.70
F9	−1	−1	−1	+1	88.51	2	0.04
F10	+1	−1	−1	+1	146.45	2	0.04
F11	−1	+1	−1	+1	98.41	2	0.04
F12	+1	+1	−1	+1	170.63	2	0.04
F13	−1	−1	+1	+1	186.46	2	0.05
F14	+1	−1	+1	+1	793.31	12	0.86
F15	−1	+1	+1	+1	198.38	2	0.06
F16	+1	+1	+1	+1	877.65	12	0.86
F16-rerun	+1	+1	+1	+1	2424.87	11	0.90

Like almost all soils, all samples contained some quartz, therefore the quartz peaks in each diffractogram were used as an internal standard for an empirical peak alignment. The 2*θ* corrections were approximated by maximising the correlation between each sample and a ‘standard quartz’ pattern [PDF 00-046-1045, ICDD, 2016] by adjusting the 2*θ* scale of the sample, before converting all data to the same 2*θ* scale using linear interpolation. This procedure empirically corrects for sample displacement and zero error simultaneously, though its primary purpose here is to ensure that peak positions between samples are internally consistent. For binning, a constant width of 5 points was used. Square-root transformations were applied to the vector of count intensities. Mean centering (Viscarra Rossel et al., 2006) was used as the method of scaling, which subtracts the mean from all values in a sample vector of XRPD count intensities. The order with which these pre-treatments were applied is reflected in the column order of Table 1.

### 2.4 Spearman and Pearson correlation coefficients

As illustrated by Gilmore et al. (2004), the Spearman correlation coefficient (*r*_*s*_) and Pearson correlation coefficient (*r*_*p*_) are sensitive to different features of XRPD data. The Pearson coefficient can be sensitive to strong Bragg peaks; with intense, aligned peaks resulting in high correlation. In contrast, the Spearman coefficient is less sensitive to major peaks, and high correlation coefficients can still arise from misaligned data. Combining these coefficients with equal weighting is therefore considered to provide a more robust measure of correlation. To combine two or more correlation coefficients, their non-linear attributes need to be accounted for (Alexander, 1990). A combined correlation matrix was therefore derived by applying a Fisher transformation to *r*_*s*_ and *r*_*p*_ (i.e. their inverse hyperbolic tangents), taking the mean of the two Fisher transformed coefficients, and then back-transforming the averaged value (i.e. the hyperbolic tangent). All correlation coefficients described hereafter relate to data subjected to this averaging procedure. The average correlation coefficients, *r*, were computed only for untreated data, and are thus denoted as *r*_untreated_ hereafter. Whilst not specifically used in the fuzzy-c-means clustering presented here, their subsequent use is intended as a simple parameter that can summarise the similarity of samples prior to pre-treatment, and aid in interpretation of the results.

### 2.5 Multivariate analysis

All methods of data analysis described hereafter were performed using the R software environment (R Core Team, 2017) unless otherwise stated. Principal component analysis (PCA) was the first multivariate method to be applied to the treated data to derive scores for the first three principal components, which together described 95.1% of total variance on average. The three PCA scores were then used in a multivariate analysis of variance (MANOVA) in order to derive an *F*-statistic from Wilks' lambda. The *F*-statistic is computed by division of the inter-group variance by the intra-group variance, therefore the objective of XRPD pre-treatment presented here is to maximise this parameter.

Non-hierarchical cluster analysis was employed using the fuzzy-c-means algorithm (Bezdek et al., 1984; Meyer et al., 2017), which was recently applied to soil spectral data since the fuzzy approach provides information on class overlaps that are inevitable in the case of soil data (Viscarra Rossel et al., 2016). Further, the partition coefficient of the fuzzy-c-means clustering statistics can be used as an objective measure to define the most suitable number of clusters (Bezdek et al., 1984; Viscarra Rossel et al., 2016). The first three PCA scores were used as inputs for fuzzy-c-means clustering, and the degree of fuzzification set to the default of 2 throughout (Meyer et al., 2017). The optimum number of clusters for each factorial treatment was derived by applying the algorithm to 11 model iterations with cluster nodes (*n*_clust_) ranging from 2 to 12, and selecting the instance with the highest partition coefficient from the clustering statistics (Bezdek et al., 1984; Viscarra Rossel et al., 2016). As a measure of cluster validity, the adjusted Rand index [*ARI*, Fraley et al., 2017] was calculated for the vector containing the classification derived from fuzzy-c-means clustering relative to the groups of the original data (i.e. 12 clusters, each comprised of 5 replicates). An adjusted Rand index of 1 would represent perfect agreement between the two vectors.

As will be described below, the objective measures of *F*, *ARI* and *n*_clust_ were used to provide recommendations for the most appropriate data pre-treatments for cluster analysis. To ensure that these recommendations were also relevant to hierarchical clustering methods, the untreated and pre-treated (i.e. all pre-treatments) data were subjected to cluster analysis using DIFFRAC.EVA software (Bruker), and the resulting dendrograms visually inspected. The implementation of hierarchical cluster analysis within DIFFRAC.EVA is based on that of PolySNAP (Barr et al., 2004a, Barr et al., 2004b, Barr et al., 2004c, Barr et al., 2004d; Gilmore et al., 2004), and uses a correlation matrix of all XRPD samples (full patterns, as outlined in Section 2.4) to compute dendrograms based on dissimilarity, *d*, where *d* = 1 − *r*.

### 2.6 Re-analysis of Bondigui samples

As will be discussed below (Section 3), two of the Bondigui measurements from the original AfSIS data collection were identified as having different count intensities and peak to background ratios to the remaining measurements in their respective sets, which affected their clustering within the dataset. These samples, belonging to the Bondigui-A and Bondigui-B sample sets, were therefore retrieved during the course of this investigation from the AfSIS soil archive and re-analysed for a sixth time using procedures described above (Section 2.2). The samples identified as having different count intensities and peak to background ratios were then substituted with the re-analysed data, and subjected to cluster analysis using the F16 pre-treatment combination (i.e. all pre-treatments; ‘F16-rerun’, Table 1).

## 3 Results and discussion

**Fig. 2:**
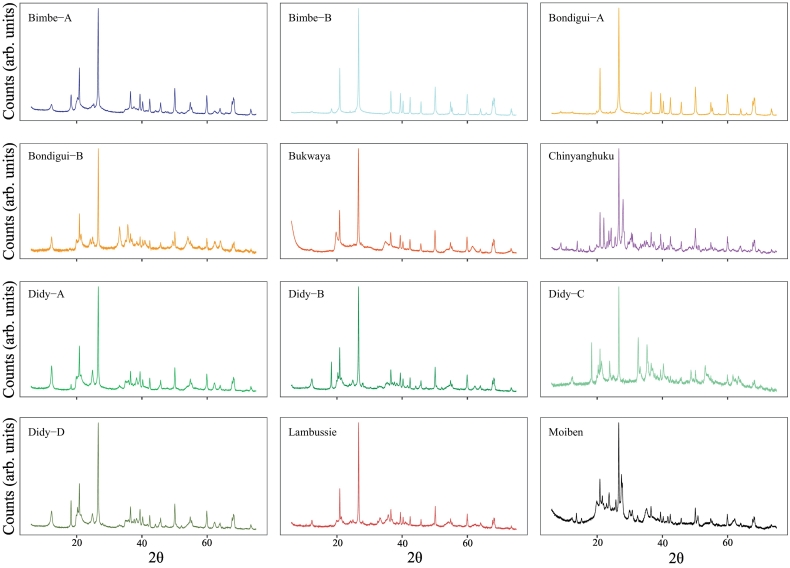
Mean diffractograms of the five replicates for each of the twelve selected soil samples, averaged at each 2*θ* measurement interval. All count intensities have been square-root transformed in order to lessen the intensity of the quartz peaks relative to the remaining mineralogy, and hence aid with comparison.

**Fig. 3:**
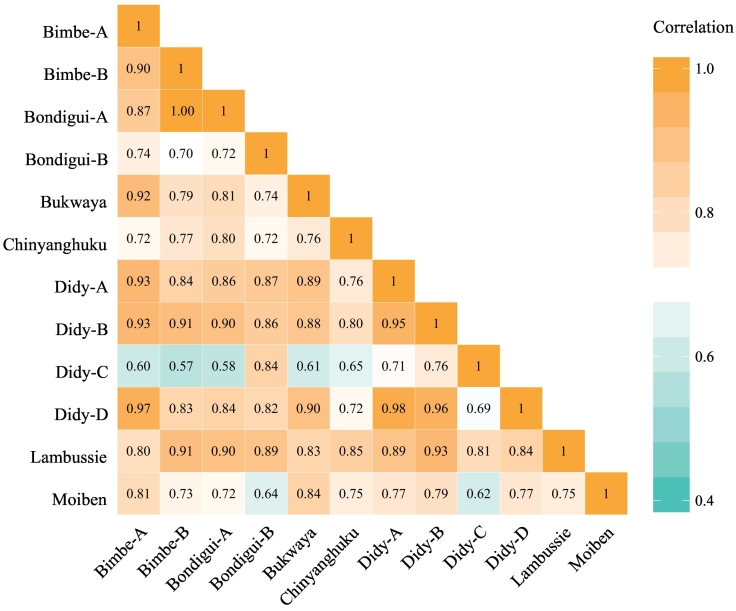
Correlation matrix (*r*_untreated_) of the 12 selected soil samples calculated on the mean diffractograms of untreated data. Correlations represent Fisher-transformed means of Spearman and Pearson correlation coefficients (see Section 2.4). (For interpretation of the references to colour in this figure legend, the reader is referred to the web version of this article.)

Providing all samples in the set of 12 presented here are mineralogically distinct from one-another (and hence produce different diffractograms), the XRPD data would be ideally expected to cluster into 12 groups, because the data are simply re-run sets of the same 12 soils. Within the selected set of 12 soils there are, however, notable similarities between some samples that can be gleaned from interpretation of the mean diffractograms (Fig. 2). To further aid in understanding this mineralogical similarity, a correlation matrix of the untreated data is also provided (Fig. 3). Together, Fig. 2, Fig. 3 highlight notable correlation for several of the selected soils. More specifically, the *r*_untreated_ between Bimbe-B and Bondigui-A is 1.00; Didy-A, Didy-B and Didy-D exhibit an average *r*_untreated_ of 0.96; and Didy-D and Bimbe-A display an *r*_untreated_ of 0.97.

**Fig. 4:**
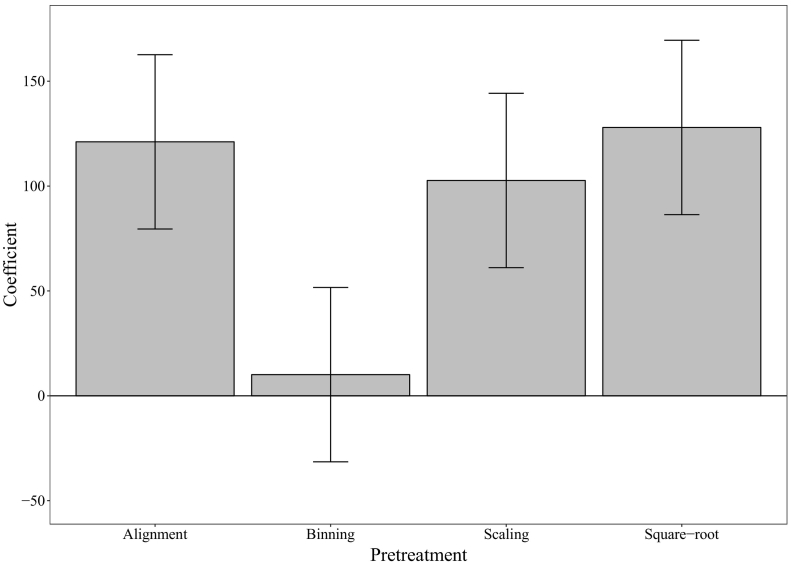
Multiple regression coefficients used as a measure of pre-treatment effects on *F* (Section 2.5).

The *F*-statistic displays substantial changes across the 16 pre-treatment combinations tested here (Table 1). To assess the overall effects that pre-treatments had on *F*, the treatment assignments (−1 or +1) were regressed against *F* using multiple linear regression (*R*^2^ = 0.69), and resulting coefficients for each pre-treatment extracted. All pre-treatments were found to increase *F* relative to the untreated data (Fig. 4). Binning displayed the lowest effect on *F*, but nevertheless its contribution remained positive (i.e. increased group variance). Although the effect of binning was not significant (*p* > 0.1), the data reduction (in this case an 80% reduction in numeric length) associated with this pre-treatment would be computationally advantageous whilst retaining the outcome of analysis in this case. Of the three remaining pre-treatments (alignment, square-root and scaling), all displayed a significant positive influence on *F* (*p* < 0.05; Fig. 4). The combination of peak alignment and square-root transformation proved to be particularly effective at increasing *F*, indicating a synergistic interaction between these pre-treatments. This highlights how small variations in peak alignment can affect inter- and intra-group variance, and that a square-root transformation is a powerful method of rescaling information relating to weakly diffracting minerals (relative to quartz in this case). When both applied, the peak alignment and square-root transformation resulted in a near 4- to 5-fold increase in *F* compared to when individually applied (with and without scaling). Lastly, scaling was also found to have a strong positive increase on *F*, resulting in a near 3-fold increase in this statistic across the pre-treatment factors compared to when it was not applied.

The number of clusters (*n*_clust_), selected from an objective measure (partition coefficient, Section 2.5), was found to be particularly dependent upon pre-treatment. No single pre-treatment was sufficient to result in *n*_*clust*_ exceeding 2. Notably, as expected from the synergistic effects on *F*, the combination of alignment and square-root transformation proved to be key for forming a more appropriate number of clusters in this case. It was only in cases where alignment and square-root transformation were applied together that *n*_clust_ exceeded 2 (increasing to 11 or 12; see F6, F8, F14 and F16, Table 1). Factors 6 and 14 (*n*_clust=11_) represent the same pre-treatments except for scaling, as do factors 8 and 16 (*n*_clust=12_). Although these pairings produce the same number of clusters, the *ARI* further highlights how scaling improves the clustering of this dataset, increasing by 0.16 in both cases.

**Fig. 5:**
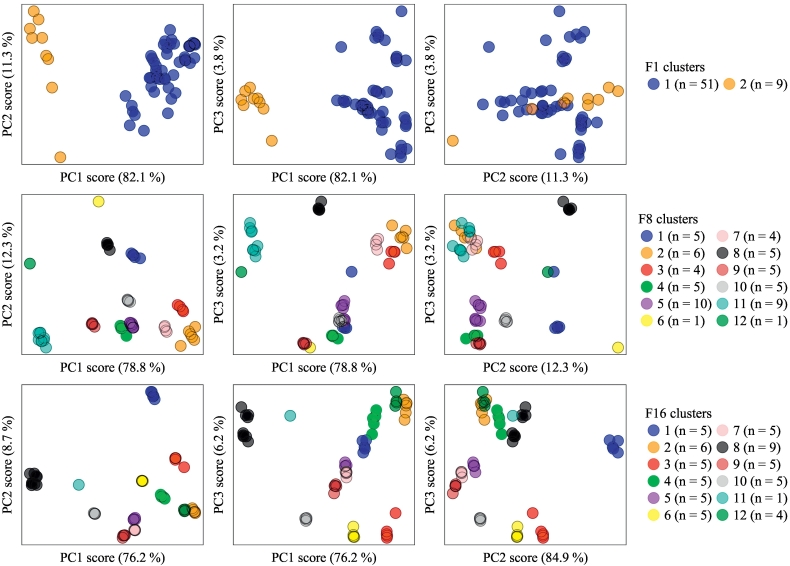
The first 3 PCA scores plotted against one-another for factorial treatments F1 (top), F8 (middle) and F16 (bottom). (For interpretation of the references to colour in this figure legend, the reader is referred to the web version of this article.)

The clustering of this data by fuzzy-c-means can be visually inspected by plotting the 3 PCA scores against one-another. For comparison, the PCA scores of F1 (no pre-treatment), F8 (alignment, binning and square-root transformation) and F16 (all pre-treatments) are provided in Fig. 5. It is evident that untreated data (i.e. F1) are difficult to classify and only two clusters (Fig. 5, top row) are observed, separated largely by the PC1 score. From this it is clearly evident that sample-independent variation of untreated data is resulting in a spread of the measurements in principal component space.

In contrast to F1, the pre-treatments associated with F8 result in a marked improvement in clustering (Fig. 5, middle row). Clusters begin to become distinguishable from one-another through a reduction in intra-group variation (largely due to peak alignment) combined with enhanced inter-group variation (resulting from the square root transformation). Nonetheless, despite improvements, the 12 clusters described by F8 display several inconsistencies. These firstly relate to clusters 6 and 12 (Fig. 5, middle row), which can be described as singletons (clusters of one measurement) and is often an indication that manual inspection is required. Cluster 6, a Moiben soil measurement (Fig. 2), should be classified within group 1 (associated with the remaining Moiben measurements), whilst cluster 12, a Bondigui-A measurement (Fig. 2), should be classified within cluster 11 (comprised of the Bondigui-A measurements as well as all Bimbe-B measurements). In addition to the two singular outliers there are further misclassifications with the F8 clustering output. First, cluster 1 contains a Bondigui-B soil sample (Fig. 2) that would ideally be clustered within cluster 2 (comprised primarily of Bondigui-B samples). As it stands, cluster 2 also contains two misclassifications - the first relating to a Lambussie sample that should belong to cluster 7, and the second relating to a Didy-C sample that should belong to cluster 3 (Fig. 2). Lastly in relation to F8 clustering, it is worth noting how Didy-A, and -B samples (Fig. 2) are grouped together within cluster 5 (Fig. 5, middle row), which reflects the relatively high correlation of their untreated measurements (*r*_untreated_=0.95; Fig. 3).

In terms of *F* and *ARI*, F16 displays the best performance of all pre-treatment combinations. This performance can also be observed from inspection of the PCA score plots and corresponding clustering (Fig. 5, bottom row).The only pre-treatment difference between F16 and F8 (described above) is the application of scaling. The result of this scaling is a notably increased *F* (Table 1) due to variances in signal intensity (predominantly resulting from the instrumental operation and collimation parameters) being minimised, which then facilitates improved fuzzy-c-means clustering (Fig. 5, bottom row). As suggested by the *ARI* value, F16's clustering is more appropriate than that provided by F8. Nonetheless two misclassification's still remain. The first relates to cluster 11, which is a sole Bondigui-A outlier that should be classified within cluster 8, where the remaining Bondigui-A measurements are clustered along with all Bimbe-B measurements. The classification of Bondigui-A and Bimbe-B into the same cluster is unsurprising given their similarity (*r*_untreated_=1.00, Fig. 2, Fig. 3). The only other misclassification from F16's clustering relates to a Bondigui-B measurement classified with cluster 2, which should belong to cluster 12 which consists of the remaining Bondigui-B measurements. It is worth noting here that despite the Didy-A and -B samples existing within the same cluster from F8's clustering (described above), the added pre-treatment of scaling is sufficient to separate the measurements from these two soils into two clusters (clusters 7 and 5, respectively; Fig. 5, bottom row). In summary, when excluding the two misclassification's from F16's clustering, the resulting output of 11 clusters, which includes the grouping of two soils with very similar mineralogies, can be considered an effective application of soil XRPD cluster analysis.

As mentioned above (Section 1.1), the effects of preferred orientation from minerals with good cleavage can be a primary factor limiting the reproducibility of XRPD measurements. None of the 4 pre-treatments tested here were designed to correct for preferred orientation, and instead care taken in sample preparation and loading by a procedure similar to that described by Zhang et al. (2003) was used to minimise the tendency for measurements to be affected by such variation. The fact that only 2 samples were misclassified when all 4 pre-treatments were applied, combined with the conclusion that this misclassification was caused by count intensities, indicates that measurement variation caused by preferred orientation, and its potential variability due to different operators performing the sample loading into the XRPD instrument, was sufficiently constrained.

**Fig. 6:**
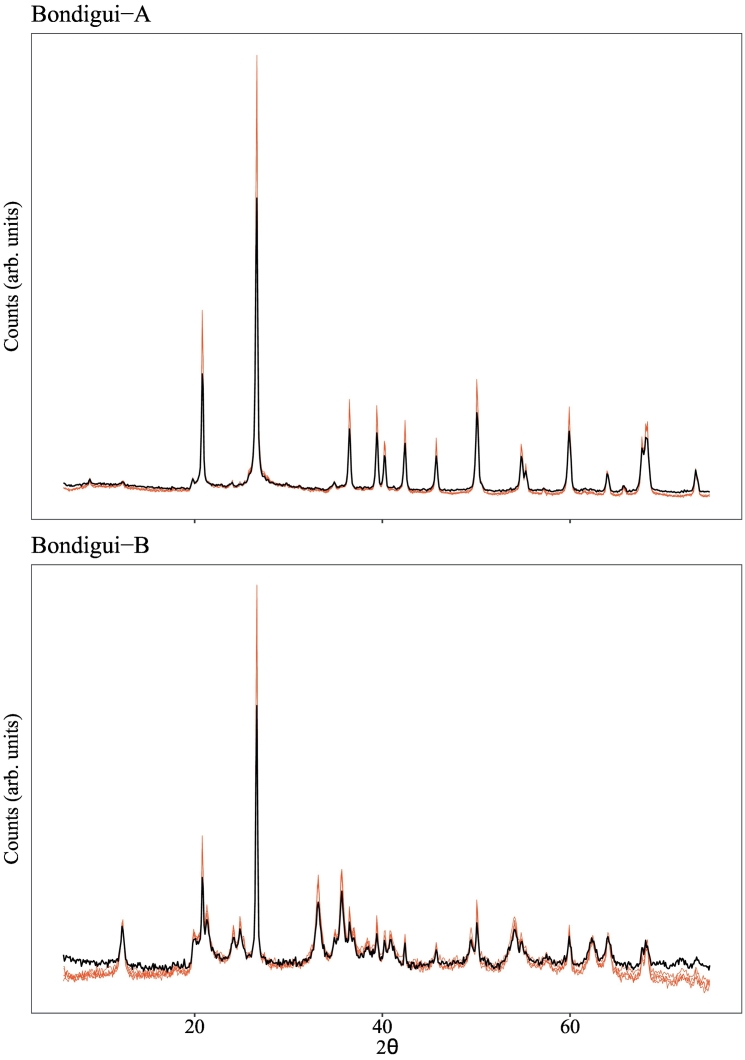
The 5 XRPD measurements of Bondigui-A (top) and Bondigui B (bottom) with all pre-treatment steps applied (i.e. F16). The misclassified measurement from each group (according to F16 clustering) is plotted in black, with the remaining 4 measurements in red. (For interpretation of the references to colour in this figure legend, the reader is referred to the web version of this article.)

It is also useful to further scrutinise the two misclassified samples that remained in F16's clustering. These relate to the Bondigui-A and Bondigui-B sets, the diffractograms of which are plotted in Fig. 6 with all pre-treatments applied. To the trained expert eye, the diffractograms within each group are clearly representative of the same sample, however the multivariate analysis applied here is more sensitive to subtle variations distributed throughout the diffractograms. In both cases it was found that the misclassified measurements are identifiable by small but significant variations in peaks heights and background intensities relative to the remaining 4 measurements. The source of this variation can be traced back to the untreated data, and their corresponding peak and background intensities. For the Bondigui-A sample, the 4 correctly classified measurements displayed similar count intensities (maximum intensity = 44,265 ± 3942) whilst that of the misclassified measurement was approximately half (maximum = 19,584). The same goes for the Bondigui-B sample, with the correctly classified measurements having maximum count intensities of 4789 ± 217, and the misclassified measurement a maximum of 2321. This misclassification in both of these cases therefore relates to count intensities and peak to background ratios, which cannot be completely accounted for using the pre-treatment steps applied here. Though the exact cause of these variations for the two Bondigui samples could not be identified, it is likely due to changes in instrument operation or collimation parameters. The finding is able to highlight the importance of ensuring comparable count intensities and peak to background ratios in soil XRPD datasets if effective cluster analysis is to be applied, particularly in the case of large datasets where assembly can span months-years.

### 3.1 Clustering of re-analysed data

**Fig. 7:**
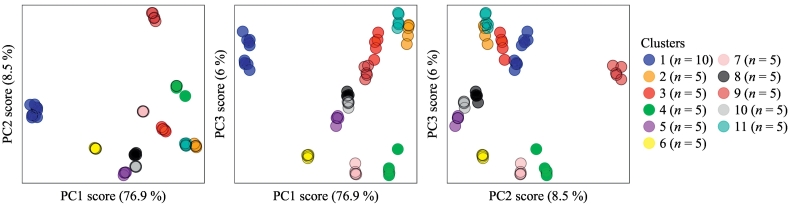
The first 3 PCA scores plotted against one-another with the identified Bondigui-A and -B samples removed and replaced with re-analysed data. Data were subject to all pre-treatments (i.e. F16). (For interpretation of the references to colour in this figure legend, the reader is referred to the web version of this article.)

After substituting the Bondigui-A and Bondigui-B samples, identified as having low count intensities and different peak to background ratios, with re-analysed data (Section 2.6), clustering of the dataset was notably improved. The improvement is reflected in the *F*-statistic, which showed a near 3-fold increase compared to the F16 results containing the original data, and also in the *ARI*, which increased to 0.90 (Table 1). Fuzzy clustering of the updated dataset yielded 11 clusters (Fig. 7). Of these clusters, 10 represented groups containing 5 correctly classified measurements, whilst the remaining cluster (cluster 1) contained 10 measurements. Cluster 1 combined all measurements from the Bondigui-A and Bimbe-B soils, which again is appropriate given the similarity of the untreated data reflected in both Fig. 2 and their high correlation (*r*_untreated_=1.00; Fig. 3). As a whole, the cluster analysis applied to re-analysed data produced groups that agree with what may be reasonably expected for the given dataset. This re-analysis again highlights how care taken to facilitate long-term instrument reproducibility is also a key step in ensuring effective cluster analysis of soil XRPD data.

### 3.2 Application of pre-treatments to hierarchical cluster analysis

The most effective pre-treatments have so far been discussed and refined in relation to cluster analysis via the fuzzy-c-means algorithm. To test whether these pre-treatments are applicable to other cluster analysis methods, the hierarchical cluster analysis implemented in DIFFRAC.EVA software (Section 2.5) was applied to the untreated and pre-treated (all pre-treatments, i.e. F16) datasets using the group average link method, which in both cases included the re-analysed Bondigui-A and Bondigui-B samples.

**Fig. 8:**
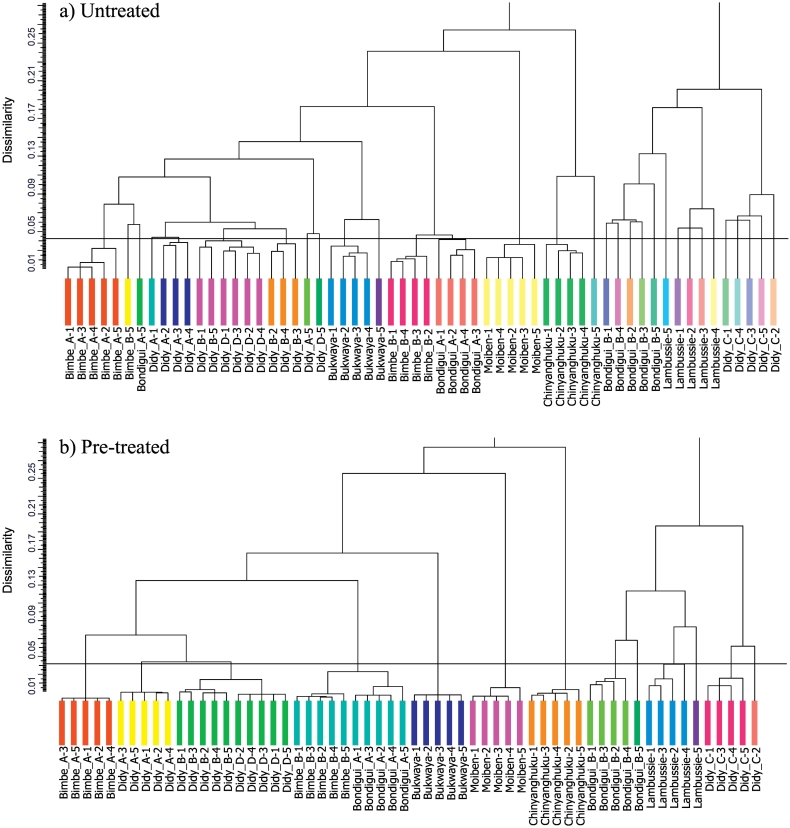
Dendrograms from hierarchical cluster analysis of a) untreated and b) pre-treated (all pre-treatments, F16) data using the group average link method. The dataset uses the re-run samples from the Bondigui-A and Bondigui-B soils (Section 2.6). The cut line in both cases is set to a dissimilarity of 0.045. (For interpretation of the references to colour in this figure legend, the reader is referred to the web version of this article.)

Selecting the appropriate number of clusters from the hierarchical cluster analysis dendrogram of untreated data (Fig. 8a) proved difficult. Setting the cut-line to a dissimilarity (*d*) of 0.045 yielded 31 clusters, 22 of which were singletons, and only two of which displayed appropriate grouping of the 5 replicates. Various cut-lines were tested on the untreated data, but none provided a suitable classification of the data into the clusters that would be expected of this dataset. In contrast, the dendrogram of the pre-treated data (Fig. 8b) displays much improved clustering. Setting the cut-line again to a dissimilarity of 0.045 yielded 13 clusters, of which 3 were singletons and the remaining were classified appropriately. Consistent with their low dissimilarity (Fig. 8b), the Didy-B and Didy-D measurements are grouped together (*d* = 0.024), as are the Bimbe-B and Bondigui-A measurements (*d* = 0.033). Upon closer inspection of the 3 singletons, each of them are close to being correctly classified with their correct clusters (Bondigui-B, Lambussie and Didy-C), and it again seems that very subtle variations in count intensities and peak to background ratios may be causing their dissimilarities to be slightly higher than the remaining 4 measurements in each set. Nevertheless the dendrograms of untreated and pre-treated data illustrate that pre-treatment by alignment, binning, square-root transformation and scaling is highly beneficial in hierarchical cluster analysis of soil XRPD data.

### 3.3 Limitations of soil XRPD cluster analysis

The sensitivity of cluster analysis to several aspects of XRPD data presented here illustrates that reproducible measurements are a key component in deriving informative clusters. A clear limitation of the approach, even with pre-treatment of the data, is that substantial variation in count intensities and peak to background ratios can detrimentally affect the clustering. Given these sensitivities it seems likely that cluster analysis of XRPD data from multiple soil datasets, where different preparation and measurement protocols are implemented on various instruments, would currently prove challenging. This therefore limits application of soil XRPD cluster analysis at present to datasets measured in a single laboratory on a single instrument. If the approach is to be applied to larger international collaborations of soil XRPD data [e.g. Viscarra Rossel et al., 2016], methods of data harmonisation will need to be carefully considered. Nevertheless, there is promising scope for the application of cluster analysis to individual soil XRPD datasets such as NSIS (Butler et al., 2018) and AfSIS (Towett et al., 2015).

## 4 Conclusions

A 2^4^ factorial design to assess the effects of four diffractogram pre-treatments has shown that peak alignment, square-root transformation, and scaling each significantly enhance the effectiveness of fuzzy-c means clustering of soil XRPD data. Further, binning the data can reduce the computational demands substantially whilst not significantly effecting the clustering, and would therefore be a useful addition for analysis of larger XRPD datasets. The combination of peak alignment and square-root transformation pre-treatments was found to have synergistic effects on the *F*-statistic. This can be interpreted as the peak alignment acting to reduce sample-independent variation, whilst the square-root transformation acts to rescale the signal of strong diffractors (in this case quartz) relative to that of minerals with weaker diffraction intensities, thus emphasising more subtle variations in mineralogical compositions of the soils. When combined, the four factors tested here resulted in accurate classification of the soil dataset into 12 clusters (using fuzzy-c-means), but two misclassifications remained that resulted from variations in count intensities and peak to background ratios relative to the remaining measurements in each set, and were presumably related to measurement problems not identified at the time of original AfSIS data collection. Upon re-analysis of these samples, the dataset clustered into 11 groups with completely appropriate classifications given that two of the soils investigated here display near perfect correlation even in the untreated data. Together this highlights how appropriate data pre-treatment combined with care taken to facilitate reproducible XRPD measurements can provide an effective basis for cluster analysis of soil XRPD data. A thorough understanding of factors that include sample-independent variation of soil XRPD data, the masking effects of strong diffractors, and data quality issues - all of which may influence the results of methods such as cluster analysis - is a prerequisite to the wider and hopefully profitable application of these techniques for exploration of soil property – soil mineralogy relationships. Ultimately the pre-treatments of alignment, binning, square-root transformation and scaling together result in significantly improved cluster analysis of XRPD data via both the fuzzy-c-means clustering algorithm and hierarchical cluster analysis.
